# Cone Photoreceptor Loss in Light-Damaged Albino Rats

**DOI:** 10.3390/ijms23073978

**Published:** 2022-04-02

**Authors:** Molly C. Benthal, Alex S. McKeown, Timothy W. Kraft

**Affiliations:** 1 Department of Optometry, University of Alabama at Birmingham, 1720 2nd Ave South, Birmingham, AL 35294, USA; molly.benthal@gmail.com; 2 Department of Vision Sciences, University of Alabama at Birmingham, 1720 2nd Ave South, Birmingham, AL 35294, USA; asmckeown@gmail.com

**Keywords:** rod, cone, photoreceptor, light damage, electroretinogram, outer nuclear layer

## Abstract

We investigated the etiology of decreased cone-driven vision in a light damage (LD) model of retinal degeneration. To induce slow, moderate degeneration, albino rats underwent low-intensity light exposure for 10 days. Electroretinography was utilized to assess physiologic function of the rod- and cone-driven retinal function in LD and control rats. Immunohistochemistry targeting cone arrestin allowed for quantification of cone density and for comparison of the decline in function. Photoreceptor loss was quantified by outer nuclear layer thickness decreases, as observed by optical coherence tomography and histology. The LD rats showed decreased rod- and cone-driven function with partial recovery 30 days after cessation of light exposure. In addition, LD rats showed decreased cone photoreceptor densities in the central retinal region compared to control rats. Our results demonstrate that the loss of cone-driven visual function induced by light damage is at least partially due to the death of cone photoreceptors.

## 1. Introduction

Retinitis pigmentosa (RP) is a broad category of inherited diseases encompassing many types of retinal degenerations. RP is the most common inherited form of degeneration and has a variety of inheritance patterns including autosomal dominant, autosomal recessive, and X-linked [1,2,3,4]. Among the diverse genetics of RP, there is a common pattern of rod degeneration preceding cone degeneration. Properly functioning cones are essential for quality of life, and thus preventing or delaying secondary cone damage is of high importance. However, the mechanisms of cone degeneration in what are classified as “rod diseases” are unclear. Thus, a more complete understanding of the degeneration profile and the survival rates of cones is of primary importance.

Studies have suggested that cone death may occur as a result of a decreased amount of Rod-derived Cone Viability Factors (RdCVFs) secreted by rod photoreceptors [5,6,7]. Cone degeneration may also result from the surrounding rod apoptosis results in disrupted ionic balance, neurotransmitter toxicity, macrophage infiltration [8], microglia polarization [9] photosensitization by porphyins [10] or a combination of these various pathways. Animal models of rod–cone degeneration include rats with a known inherited retinal degeneration, transgenic mice, and light damage (LD) in pigmented and albino animals [11,12,13,14]. Both acute (minutes to hours) and longer-term (days to weeks) light exposure can cause different forms of stress and retinal degeneration [15]. Pigmented animals require much higher levels of light exposure and may be less representative of chronic or slowly progressing diseases [16]. Due to the complex genetics of RP mentioned previously, the study of models with broader patterns of retinal damage such as the LD model can provide valuable comparisons to human conditions, although some authors argue for the use of pigmented animals [17]. LD can be acute (intense light, brief damage) or sustained (lower light levels, longer exposures), with each technique offering different insights on neuroprotection and degeneration [18]. Recent studies have investigated ranges in the visible spectrum and have demonstrated shorter wavelengths to be the most phototoxic [10,15]. The study presented here used sustained LD, as rats were exposed to continuous, white light of a moderate intensity (325 lux) for multiple days, matching a parallel study of visual behavior [19].

There is clear photoreceptor loss in sustained LD, but whether rods or cones are more susceptible to LD is a controversial topic. Early studies showed that rats succeeded at tasks of pattern discriminations after light damage despite significant rod loss, suggesting that spatial resolution tasks were mediated by the surviving cones [20,21,22]. Behavioral and electrical testing of rod-driven function appears resilient, whereas cone-driven function shows a deficit [19]. Other studies have demonstrated a decrease in both rod- and cone-driven function through reductions in both light- and dark-adapted electroretinography (ERG) b-wave amplitudes and thresholds [23]. Recently, Riccitelli and colleagues thoroughly examined the light damage effects of brief (12–24 h) 1000 lux exposures on retinal morphology, anatomy, and function, as well as molecular signatures [24]. They found that functional losses were detectable prior to anatomical changes and that loss of 75% of cones resulted in an immediate loss of photopic b-waves of similar magnitude. They also found that substantial recovery was possible, although it was incomplete and transient on the scale of months. In this study, we sought to contribute to the understanding of cone degenerations in a mild light damage model. We confirmed photoreceptor loss through functional and histological measurements and found that there is significant cone loss in the central portions of the LD retinas.

## 2. Results

### 2.1. Electroretinography

**Figure 1:**
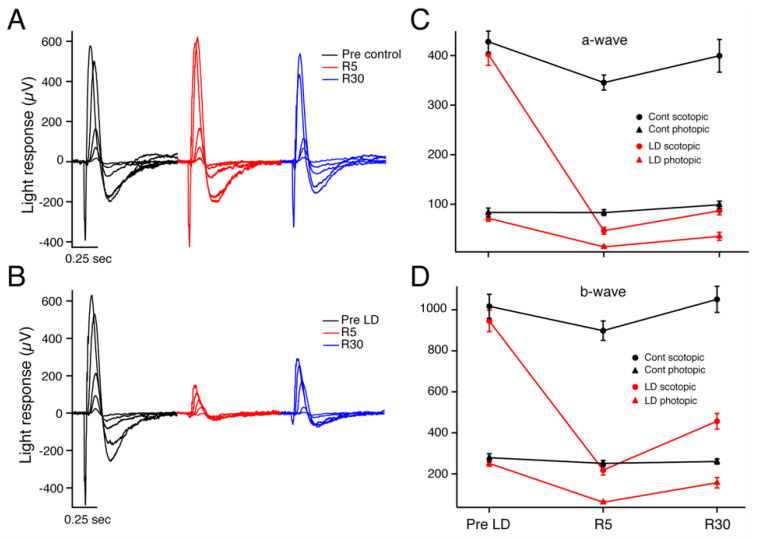
ERG representative intensity response series from a control animal (**A**) and an experimental light-damaged 47-day-old Sprague–Dawley albino rat (**B**) before light damage (black traces), 5 days after recovery from light damage or sham light damage (red traces), and at 30 days after recovery from light damage or sham light damage (blue traces). Individual traces represent average response from 3–20 repeated stimuli. Averaged maximum ERG response amplitudes for the a-wave (**C**) and b-waves (**D**) are given. Dark-adapted (rod-driven, circles) and light-adapted (cone-driven, triangles) a-wave amplitudes of LD animals (red) are decreased at R5 and partly recovered at R30, while the control animal results (black) are unchanged. (**D**) LD b-wave response amplitudes (red traces) show a similar pattern but with greater recovery between R5 and R30 timepoints. Error bars = mean + SEM.

Dark-adapted, light-adapted, and flicker ERGs were recorded for light-damaged (LD) and control groups before LD (*n* = 29), 5 days after LD (*n* = 27), and 30 days after LD (*n* = 26). All animals used were Sprague–Dawley albino rats, and litters were split randomly into experimental (light exposed, LD) vs. control (normal cyclic light throughout) animals. Figure 1 depicts an intensity response series of dark-adapted flash ERG recordings in an individual control and LD rat before light damage (black traces), on recovery day 5 (R5) (red traces) and thirty days after light-damage regimen (R30) (blue traces) for a control animal (Figure 1A) and an LD animal (Figure 1B). As demonstrated in this specific example, the average a- and b-wave amplitudes of the LD rats were significantly reduced after light damage at R5, and both showed partial recovery after 30 days of recovery (Figure 1C,D). At R5, the LD average dark-adapted (rod-driven) a-wave maximum amplitude decreased dramatically to only 12.5 ± 2.0% (*n* = 14) of the Pre-LD level, and the b-wave maximum amplitude was also strongly reduced to 24.2 ± 3.2% (*n* = 14) of its control value. By R30, the LD scotopic a-wave maximum amplitude had recovered to 23.0 ± 3.4% (*n* = 13) of its pre-LD value. The b-wave also recovered, and to a greater extent of 50 ± 6.5% (*n* = 13) of its pre-LD value.

The photopic (cone-driven) ERG at R5 a-wave maximum amplitude average decreased to 22 ± 3.8% (*n* = 14) of its Pre-LD level, and the b-wave maximum amplitude decreased about the same, to 25 ± 2.8% (*n* = 14). After a 30-day recovery period, the photopic a-wave amplitudes had improved but were still significantly reduced from pre-LD levels (30.8 ± 5.0% (*n* = 11) *p* < 0.01). The LD average light-adapted b-wave maximum amplitude more than doubled its R5 amplitude but was still reduced to 56 ± 8.2% (*n* = 13) of its pre-LD value. There was no significant difference in the average dark-adapted and light-adapted a- and b-wave amplitudes of control rats measured at the same time points. Table 1 lists the average maximum a- and b-wave amplitudes for scotopic and photopic ERGs performed (Figure 1C,D).

The threshold sensitivity for LD animals, which was measured by the amplitude of a dim light response, was found to have decreased significantly at R5 and then recovered somewhat by R30. The sensitivity of the entire intensity vs. response function was assessed by measuring the flash intensity required to evoke a half-maximum response (I_1/2_). As expected, at R5, the I_1/2_ for LD animals was significantly greater than that of control animals (*p* < 0.05) indicating reduced overall sensitivity. The threshold sensitivity and I_1/2_ values were not affected in control animals. Table 1 lists the average sensitivity and I_1/2_ values for control and light-damaged rats. 

**Figure 2:**
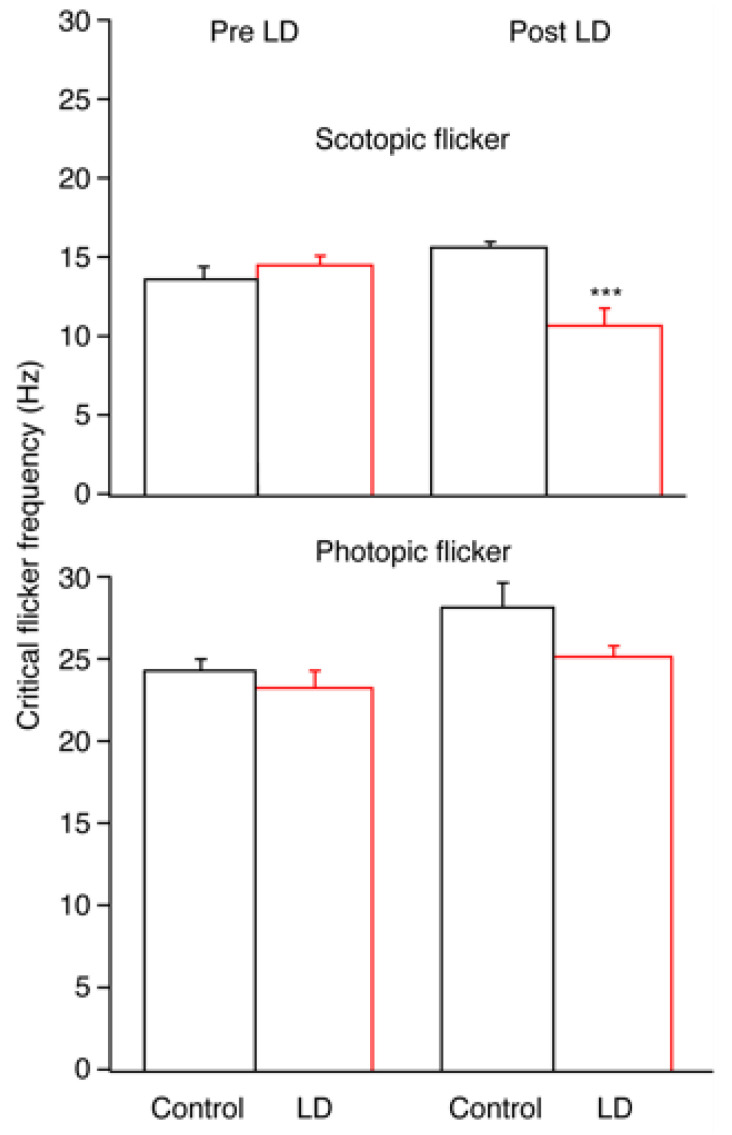
Flicker thresholds (CFF) measured under scotopic (upper graph) and photopic (lower graph) conditions were measured before (Pre LD) and after (post LD) a 10-day continuous exposure to a low-level light damage (325 lux) regimen. The LD group (red bars) was significantly reduced compared to the controls (black bars) only under scotopic conditions 30 d after light damage. Mean ± SEM. *** *p* < 0.001.

A measure of dynamic retinal function was tested by measuring the ERG critical flicker frequency (CFF) under rod-driven (scotopic) and cone-driven (photopic) lighting conditions. Scotopic ERG CFF was greatly reduced in LD animals compared to control animals, whereas under photopic conditions, the CFF values were similar. Dark-adapted (scotopic) CFF in LD animals was decreased by about 33% at R30 (*p* < 0.01), whereas light-adapted (photopic) CFF remained stable at R30. At R30, the LD average dark-adapted CFF had decreased to 75.0% ± 7.5% (*n* = 6) of the pre-LD value and was only 2/3 of the scotopic CFF of the control group at the same time point. Figure 2 demonstrates the changes in scotopic and photopic CFF values.

### 2.2. Optical Coherence Tomography

**Figure 3:**
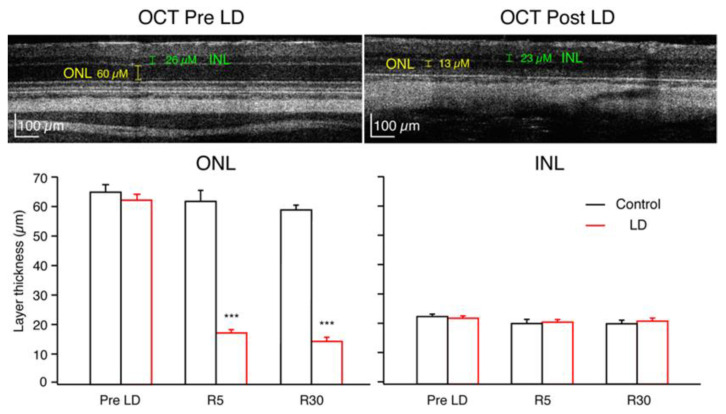
High-resolution spectral domain OCT image of an LD rat, upper left image at PN48, before light damage. ONL thickness was 60 μM, and INL thickness was 26 μM. Upper right image from the same rat at age PN89, 30 days after recovery from light damage. ONL thickness was measured to be 13 μM, and INL was 23 μM. Graphs below show population averages (±SEM) for control (black) and LD (red) groups. (*** *p* < 0.001).

Figure 3 depicts OCT images of a rat retina before light damage and at R30. As demonstrated in this specific example, the average ONL thicknesses of the LD rats was significantly reduced after LD, while the controls were not affected. At R5, the LD average ONL thickness decreased to 28.5% ± 1.8% (*n* = 12) of the Pre-LD level, and by R30, it was 23.4% ± 1.6% (*n* = 9) of the Pre-LD value (Figure 3). There were no significant differences between INL thickness measures in LD rats compared to control rats at any time point. 

### 2.3. Immunohistochemistry

**Figure 4:**
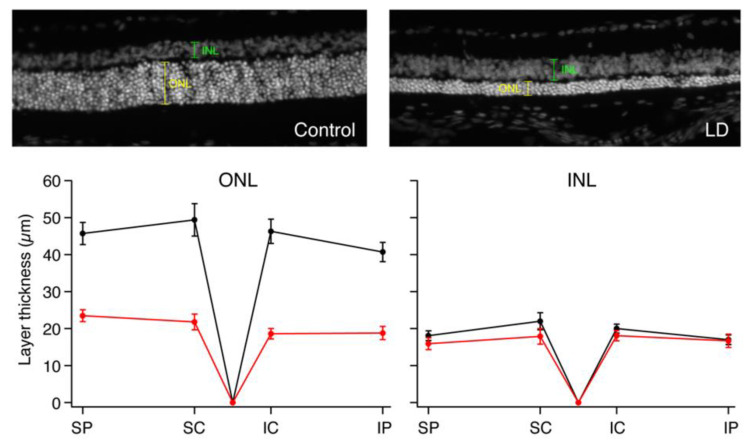
Histological differences between ONL and INL thicknesses in control versus LD rats. Photograph of 10 μM thick frozen retinal section stained with DAPI in an individual control rat (left) and LD rat (right). Graphs show the average ONL and INL thicknesses in the four quadrants measured: dorsal peripheral (SP), dorsal central (SC), ventral central (IC) and ventral peripheral (IP) quadrants of control and LD rats measured from frozen retinal sections stained with DAPI.

**Figure 5:**
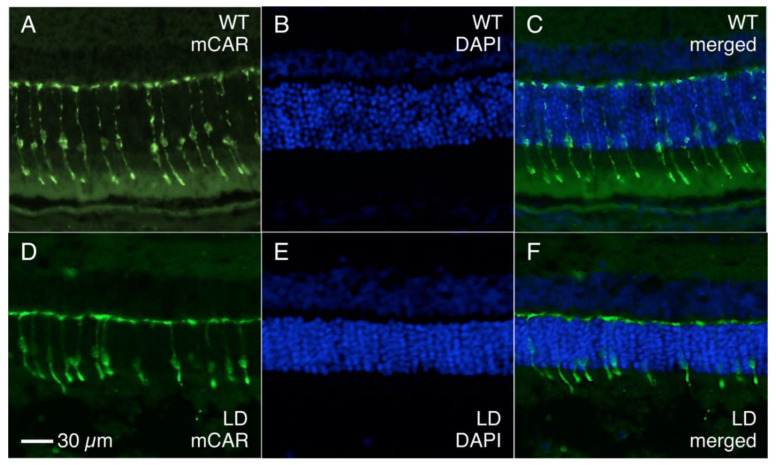
Fluorescent photographs of 10 μM frozen retinal sections from control and LD rats stained with mCAR and DAPI. (**A**,**D**) Images taken with an Alexa filter in place to show vibrant cone photoreceptor staining of mCAR. Cone densities were counted from these images for each animal. (**B**,**E**) Cell nuclei in the ONL and INL stained with DAPI. (**C**,**F**) Merged images of Alexa and DAPI filters. The control and LD animals depicted were sacrificed on R32 and R30, respectively.

**Figure 6:**
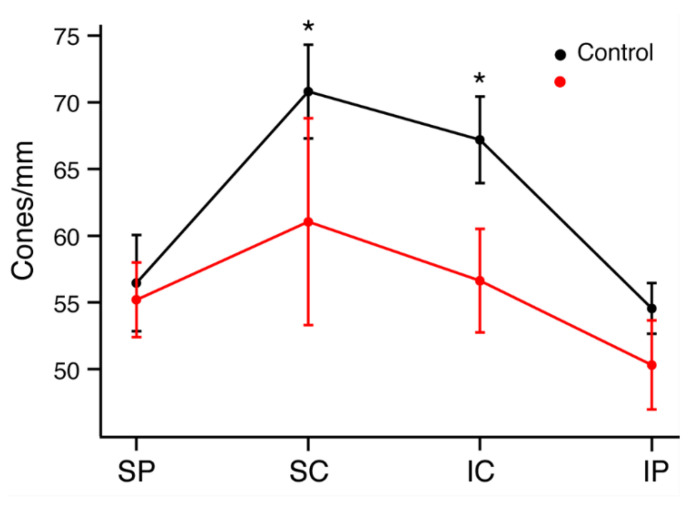
Average cone photoreceptor densities of control and LD rats by region. Retinas were divided into dorsal peripheral (SP), dorsal central (SC), ventral central (IC) and ventral peripheral (IP) quadrants. * *p* < 0.05.

To quantify and localize the cone photoreceptors, retinal sections from each rat were stained with an antibody for cone arrestin (mCAR). Stained cones fluoresced vibrantly, allowing for easy differentiation of individual cones for measuring cone density. Figure S1 shows the sectioning method used to group retinal regions and indicates the total number of retinal quadrants with bright, uniform photoreceptor staining from which cone counts were attained (*n* = 4–6 LD; *n* = 6–10 control). Any quadrant that included irregular or faint staining was not included in cone density measures. ONL and INL thicknesses were also measured in all photographs regardless of quality of cone photoreceptor staining, and there was significant ONL thinning in the LD retinas when compared to WT (Figure 4). The relationship between rod loss and ONL thickness has been documented [25], and thus cell nuclei counts were not performed. Average ONL thicknesses were 49.7% smaller in peripheral retina and 57.3% smaller in central retina in LD animals compared to controls. There was no significant difference between INL thickness measures in LD rats compared to control rats. Figure 5A–E shows composite images taken from control and LD rats with uniform cone photoreceptor staining. When the cones were counted, LD rats showed decreased cone densities in the central regions when compared to the controls. Central cone densities were 15.6% lower in the LD rats than the control rats (Figure 6, *p* < 0.05). Peripheral cone densities were not significantly different between control and experimental groups. 

## 3. Discussion

The goal of this experiment was to characterize the etiology of decreased cone-driven vision in the light damage model of retinal degenerations. Because most retinal degenerations are typically categorized as “rod” diseases, fewer efforts have been directed toward the effects of light damage on the cone photoreceptors in chronic light exposure models, although recent studies have looked at focal, blue light and LED damage to cones [15,26]. By analyzing and comparing visual functional changes (via ERG), in vivo histology (via OCT), and in vitro histology (via immunohistochemistry), our study provides an investigation of cone density changes as well as rod loss resulting from low-level, long-duration light damage.

### 3.1. Electrophysiologic Assessments of Visual Function

We have demonstrated severe rod and cone functional deficits induced by relatively low levels of LD, which we feel begins to mimic the chronic conditions that lead to retinal degeneration in human disease that may arise from a large number of diseases. There was progressive thinning of the ONL between recovery day 5 and day 30 that likely indicates a slow clearance of non-functional photoreceptor cell bodies. We did not specifically look for classical markers of apoptosis, such as tunnel, but this type of cell clearance would be expected. Despite the continued ONL thinning, there was significant recovery of the a- and b-waves, indicating that there may be some plasticity in the signaling of the retina. This type of plasticity or recovery has been noted before in rodent models of degeneration [14,27], and bipolar cell sprouting is a potential contributing factor [28,29]. An electron microscopic examination of the outer plexiform layer could confirm synaptic remodeling. Both the scotopic (rod-driven) and the photopic (cone-driven) responses showed partial recovery by 30 days post-light damage. As a nocturnal animal, the rat has a much higher proportion of rod photoreceptors than cones, about 20:1, yet even with cone losses, we found persistent cone signals recordable with the photopic ERG. The substantial synaptic gain between cones and cone bipolar cells may explain the persistence of these signals [30].

Additionally, by recovery day 30, the photoexposed animals had significant improvements in sensitivity when compared to R5. At R5, the max scotopic and photopic b-waves decreased to 25% and 24%, respectively. However, by R30, the max scotopic b-wave recovered to 50% of pre-light-exposed levels, while the max photopic b-wave only recovered to 44% of pre-light-exposed levels. It appears that rod-driven visual function is more resilient to degenerative stresses and photoreceptor loss. A 15% cone loss in the central retina results in permanent loss of ~55% of the max photopic b-wave, while loss of almost 60% of the rods results in a loss of only ~50% of the max photopic b-wave. Because the light-damage regimen lasted 10 days, some of the changes in a-wave amplitude and recovery could be ascribed to photostasis, the process of reductions or increases in the lengths of the outer segments of the rod and cone photoreceptors [31]. These OS length changes could also partially explain the changes in ERG sensitivity that we measured. However, the relative difference in the proportions of loss of cell numbers vs. loss of function cannot be explained by photostasis alone. The electrophysiological response of surviving rods in retinas under degenerative stress are reportedly altered [32] and could also be a contributing factor.

Scotopic CFF declined after light damage (at both R5 and R30), but photopic CFF remained steady at each time points. This result is surprising because the light-adapted a-wave amplitude (measuring cone function) decreased by 44% following light damage (R30). A previous study demonstrated a significant decrease in photopic behavioral CFF at 6 days after light damage, yet photopic ERG CFF was not reduced at the same time point [19]. A possible explanation is the difference in testing by flash ERG versus CFF. Flash ERG measures the maximum possible cone-driven voltage attainable via cone circuits. CFF, however, measures a threshold response, which is a measure of cone circuitry at the opposite end of the intensity response range. The criterion response set for CFF was 4.2 log units μV^2^*Hz (this value was chosen because it is representative of the RMS noise of our flicker recordings). If the criterion of detection of flicker was set higher or lower, a more pronounced deficit may have emerged. In addition, because CFF measures the threshold required to evoke a response, it is a better gauge of the remaining cone function following light damage. Full-field flash ERG, conversely, is a better measure of the decrease in maximal possible responses following degeneration.

### 3.2. In Vivo and In Vitro Imaging of Retinal Layers

Obtaining in vivo measurements of ONL thicknesses was a unique approach for gauging the progress of LD-induced retinal degeneration. Our results reveal that decreases in ONL thickness due to light damage are not transient. Multiple studies, including ours, demonstrate recovery in physiologic function of the retina following 2–4 weeks of recovery from damaging light exposure; however, earlier comparisons of visual function to ONL thicknesses were typically made after the time of sacrifice when histology was performed. In addition, despite the large decrease in ONL thickness at R30, ERG measures showed recovery in rod-driven substrates for scotopic vision. Therefore, the rod system may be undergoing synaptic remodeling in order to recover visual function. Additionally, because the ONL does not continue to decrease by R30, our results show that the 10 days of light damage do not trigger an unstoppable cascade for photoreceptor death as seen in genetic models of retinal degeneration. Thus, the recovery phase in our model represents a unique opportunity to measure the resilience of the retinal circuitry and future potential therapeutic interventions.

The data collected revealed a small discrepancy between the raw values of ONL and INL thicknesses measured via OCT versus those measured via immunohistochemistry. For the control group of animals, the average R30 ONL thickness measured in OCT was 59.6 ± 1.5 μM, while the average thickness measured from frozen sections was 42.4 ± 2.5 μM. Similarly, the average control R30 INL thickness was 20 ± 1.0 μM from OCT and 18 ± 1.3 μM from frozen sections. Thus, average OCT measurements of ONL and INL were approximately 28% and 10% greater than in vitro measurements. A study by Quihong L. et al. (2001) [33] documented a similar phenomenon where OCT retinal thickness measures were greater than those measured by histology; however, when the data were normalized, there were no significant differences between the two methods. One explanation for the smaller values measured via histological sections is tissue shrinkage and dehydration during the enucleation and fixation processes; additionally, it has been demonstrated that OCT can introduce measurement bias [34].

Our visualizing/counting of cones was made using cone arrestin. It may be of interest to stain for specific opsins to see if the cone types were impacted differently. Were s-cones at higher risk? Or are all cone types impacted similarly? Our distribution of cone losses suggests a uniform loss with respect to a dorsal nasal axis, while the cone distribution of cone subtypes in albino and pigmented animals is known to be highly polarized [35,36].

The idea of a slow process of continuous physiological stress as introduced by 10 days of low levels of constant light presents significant advantages as a model for chronic retinal disease in humans, wherein damage accrues over years and decades of life. It has the advantage of being titratable so as not to introduce a massive inflammatory response and photochemical damage through the creation of reactive oxygen species (ROS) [37] allowing opportunities for protective therapies [38]. Many human retinal diseases present in the fourth decade and beyond, and thus the sustainability of retinal signaling and the opportunity for intervention in this type of animal model offers great opportunity to test interventions before, during, and after the degenerative stress. This approach is also relevant for AMD, which arises from a lifetime of photoreceptor activity, oxidative stress, and metabolic challenge and which attacks the rods first but ultimately results in catastrophic loss of cone-dependent vision [39], excellently reviews by Nigayle et al (2022) [40].

## 4. Materials and Methods

The ARVO Statement for the Use of Animals in Ophthalmic Vision Research was used as a guide for the handling of all animals in this experiment, and all procedures were approved by the University of Alabama at Birmingham’s IACUC. Sprague–Dawley albino rats were bred and housed in a cyclic 12 h light/dark room (10–18 lux) in UAB’s animal housing facility. The experimental paradigm was as follows: pre-light-exposed, at post-natal day (PN), 40–42 litters of rats underwent both ERG and OCT measurements. The siblings were then randomly sorted into LD and control groups. At PN45, the experimental group began their 10-day regimen of light damage, while the control group remained under normal cyclic lighting conditions. ERGs and OCTs were again performed on all of the rats at 5 days of recovery from light damage (R5) and at 30 days of recovery (R30). After the final round of ERGs and OCTs were performed, the rats were sacrificed, and their eyes were enucleated, fixed, cryosectioned, and stained with fluorescent antibodies for mouse cone arrestin.

### 4.1. Light Damage

At PN day 45, rats from the experimental group were placed in a room with continuous 325 ± 2.5 lux fluorescent lighting for 10 consecutive days in order to induce photoreceptor damage. The light intensity was measured at 6 places within the cages. Two rats were housed in one cage, separated by a steel divider splitting the cage into equal halves, thus preventing them from hiding under any shadows, including that of their cagemates. This system was utilized to help promote social interaction between the animals and to reduce the stress of constant light exposure. Each cage contained one water bottle per animal, and food was placed on the floor of the cage in order to maintain uniform lighting, which was measured in six locations in each cage. A single 32-watt fluorescent light bulb (T8, Philips, 2950 Lumens, Color Rendering Index 85, Color Temperature 3000 K) was hung four feet above the bottom floor of the animal cages. Illumination levels were measured immediately before and after the 10 days of light damage with a Digital Photometer/Radiometer J-6511 (Tektronix Corporation, Richardson, TX, USA); the apparatus had room for three such cages, which were rotated in location and orientation each day. The total cohort for this experiment was 29. Twenty-six animals survived to day R30, including 13 control rats and 13 light-damaged rats. Three animals died before R30 due to complications from anesthesia administration.

### 4.2. Electroretinography

ERGs were recorded on the left eye of each animal before LD, 5 days after recovery from the photodamage (R5), and about 30 days after recovery from photodamage (R30) Marmor [41,42]. Rats were dark-adapted overnight before ERG recordings. On the day of recording, the rats were sedated with 3–5% isoflurane and then anesthetized with an intraperitoneal injection of xylazine (9.09 mg/kg) and ketamine (90.9 mg/kg). Both corneas were anesthetized with 0.5% proparacaine hydrochloride, and the left pupil was dilated with a topical mixture of 2.5% phenylephrine hydrochloride and 1% tropicamide. The rat was then placed on a heating pad (~37 °C, Deltaphase isolthermal heat pad, Braintree Scientific, Braintree, MA, USA) inside a Faraday cage, and the upper teeth were positioned over a bite-bar in order to stabilize the head. A drop of Goniosol (2.5% hydroxymethylcellulose) was placed on the corneas. A platinum wire recording electrode, mounted on a fiber optic cable, was placed on the left cornea, and a gold wire reference electrode was placed on the right cornea. The faraday cage was then closed with an electrically grounded curtain. Light stimuli consisted of 2 ms flashes of 505 nm light (Bandpass filtered ± 17 nm), and stimuli were attenuated with combinations of neutral density filters. Light responses were amplified (CP122W; Astro-med West Warwick, RI USA.; DC 300 Hz) and digitized at 2 KHz (Real-Time PXI Computer; National Instruments, Austin TX, USA). Next, light-adapted full-field flash ERGs were recorded in order to isolate cone-driven responses. A bright light stimulus of 500 nm (6.6 log-photon μm^−2^) was flashed in the presence of a rod-saturating background light (about 5000 photons μm^−2^s^−1^ incident upon the cornea). Flicker ERG was also performed in order to separate rod- and cone-driven responses. A ferro-electric liquid crystal shutter (LV050, Displaytech, Inc. Boulder, CO, USA) was used to produce flicker by sinusoidally modulating stimulus intensity. Stimulus frequencies were recorded at 1, 2, 4, 5, 10, 16, 20, 25, 32, and 40 Hz. Experimental runs for each temporal frequency were sampled at 4000 KHz. Two mean light intensities were used: one dim flicker was used to assess rod-driven responses, and a second brighter flicker was used to isolate cone-driven responses. Light sources were calibrated daily using a photometer (model 350 linear/log optometer; Graseby Optronics, Elgin, IL, USA). Calibration values were used to convert energy into stimulus strength (photons/μm^2^ incident upon the cornea).

$$R=R_{\max}\left[1-\exp\left(-\frac{i}{10^{k}}\right)\right]$$

After plotting the amplitude vs. intensity data for both the a- and b-wave, the results were fitted with a modified Michaelis function of the form: where *R* = response, *R*_max_ = maximum response, *i* = log intensity, and *k* = log of I_1/2._ For flicker ERG analysis, the initial on-transient of the response was ignored, and a fast Fourier transform was performed on the final 4.5 s of data at each stimulus frequency. The power of each response at the stimulus or driving frequency was calculated by subtracting the average noise from the total area under the peak of the transform. This noise was equal to the average area to the left and to the right of the peak under a distance that was equal to the width of the peak. A criterion of a 1.65× or greater signal-to-noise ratio was set for including a response as a signal. The log_10_ responses at each frequency were then fit with a line to determine electrophysiological CFF using 4.2 log units μV^2^*Hz as the criterion voltage (Supplementary Materials) for example of full-field and flicker ERG analyses).

### 4.3. Optical Coherence Tomography

Immediately following all ERG recordings, while the rats were still anesthetized, OCTs were performed on the same eye (left) that was stimulated during ERG. The pupil remained dilated from the ERG recording, and artificial tears were applied to the eye every few minutes to decrease corneal dehydration and improve image quality. The animal was restrained in a mounting tube, and the eye was lined up with the recording probe of the high-resolution spectral domain OCT (Bioptigen, InVivoVue^TM^ Diver, Release 2.0; Duke, NC, USA). Using the fundus camera for guidance, precise alignment for acquiring a high-quality OCT image was achieved. The reference arm setting for rat retinas was approximately 1117. Two individual scans were recorded for each animal: one with a 3.2 × 3.2 mm^2^ area centered around the optic nerve head, and one with a 1.6 × 1.6 mm^2^ area centered around the optic nerve head. Scale bars were used to manually measure outer and inner nuclear layer thicknesses.

### 4.4. Histology

After ERG and OCT recordings on day R30, animals were sacrificed according to the Animal Resource Program (ARP) protocols approved by UAB’s Institutional Animal Care and Use Committee (IACUC). The eyes were immediately enucleated and placed into 2% paraformaldehyde fixative. After fixing for approximately 10 min, the cornea was removed to allow for better penetration of fixative and buffers. Eyes were then placed back in fixative overnight at 4 °C. The following morning, the eyes were placed into a sequence of: 0.1 M phosphate buffer, 30% sucrose solution, and an embedding medium. Eyes were oriented in a cryosection mold using the optic nerve and the long posterior ciliary artery (LPCA) as a marker of the ventral retina. The eye was frozen in place in the cryosection mold, and 10 μM thick frozen sections of tissue tangential to the LPCA were sliced and mounted onto slides. Tissue was collected from approximately 500 μM medial through 500 μM lateral to the optic nerve head (ONH) (Figure S1).

### 4.5. Immunohistochemistry

A cross section of retina that intersected the optic nerve head from each animal was stained with a 1° antibody for mouse cone arrestin (mCAR), a 2° antibody with an Alexa-488 fluorophore, and DAPI. A control slide for each animal was only stained with the 2° antibody and DAPI. Within one week of antibody staining, fluorescence photography was performed (Zeiss Axioplan2 Imaging, Serien-NR 3511001441, EXFO X-Cite 120 Fluorescence illumination system, AxioCam HRm camera, AxioVision Rel. 4.6 software; Zeiss USA, Hebron, KY, USA). Because each section was 10 μM thick, Z-stack photos were taken in order to capture 2 μM thick vertically layered images of tissue within each frame. Composite photographs of the retina were captured with both the Alexa and DAPI filter individually. Cone counts, retina lengths, ONL thicknesses and INL thicknesses were all measured from such Z-stacks. The four regions of the cone that stained from brightest to dimmest were the pedicle, outer segment/inner segment, nucleus, and axon. The criterion for including a cell in the cone count was clear visibility of two of the four regions. A second counter who was unaware of the animal type or of the initial count independently verified sample counts. The length of the retina, measured along the IPL, was also gathered from each composite photograph (See Figure S2). The full length of the retina was measured and divided into 4 quadrants of equal length: dorsal peripheral (SP), dorsal central (SC), ventral central (IC), and ventral peripheral (IP). The totals cones found in a complete section numbered approximately 400–650 per retina, minimizing any effect of counting errors. The number of cones in each quadrant was summed, giving a total number of cones/mm. Three measures of ONL and INL thickness were averaged for each composite photograph (about 400 μM of retinal length per photograph), and these values were averaged within each quadrant of the retina. Further details of the immunohistochemistry are given in the Supplementary Material and in Clark (2013) [43].

### 4.6. Statistical Analysis

A two-tailed *t* test was used to compare paired data from control and light-exposed animals for ERGs and cone densities. A two-way analysis of variance was used to compare regions of cone density in control and light-exposed animals. The significance level used for all analysis was *p* < 0.05. Data are presented as mean ± SEM.

## 5. Conclusions

Cone densities were decreased following light damage in the central retinal regions, but the decrease in photoreceptors was not as substantial as the decline in physiological function of the retina. Cone photoreceptors have a robust range of light adaptation and would likely not be stressed by constant light; thus, other mechanisms are likely contributing to the significant declines in cone-driven function induced by our 10-day chronic light exposure. The retinal plasticity demonstrated by the recovery of the ERG responses and the relatively small loss of cone photoreceptors provides incentive to continue searching for multiple treatments for patients with retinal degenerations.

## Figures and Tables

**Table 1:** ERG Results. ERG measures were taken before light damage (Pre-LD), and 5 and 30 days into the recovery period (R5 and R30, respectively). Reported below are the maxima attained under dark- and light-adapted conditions. (mean ± SEM).

	Dark Adapted Amplitude (μV)		Light Adapted Amplitude (μV)	
	a-Wave	b-Wave	a-Wave	b-Wave
**Pre-LD**				
Control	428 ± 21	1017 ± 58	83.5 ± 8.7	279 ± 19
LD	403 ± 23	945 ± 52	71.7 ± 6.2	250 ± 14
*p* value n.s.				
**R5**				
Control	345 ± 15	898 ± 47	83.2 ± 6.0	251.0 ± 4.0
LD	46.6 ± 7.1	218 ± 25	13.9 ± 2.2	61.0 ± 6.1
^1^*p* < 0.001				
**R30**				
Control ^2^	400 ± 33	1051 ± 64	98.9 ± 7.3	260 ± 13
LD	86.7 ± 8.3	456 ± 38	30.8 ± 5.0	157 ± 25
	*p* < 0.001	*p* < 0.001	*p* < 0.01	*p* < 0.01

^1^ Control vs. R5 *p* < 0.001 for all comparisons; ^2^ R30 Comparisons are to R30 control which was n.s. compared to Pre-LD control.

## Supplementary Materials

The following supporting information can be downloaded at: https://www.mdpi.com/article/10.3390/ijms23073978/s1.
