# Supplementary material for: Cone Photoreceptor Loss in Light-Damaged Albino Rats
Source: Int J Mol Sci. 2022 Apr 2;23(7):3978. doi: 10.3390/ijms23073978 (PMC8999964; doi:10.3390/ijms23073978)
Supplement: Supplementary file 1 [file ijms-23-03978-s001.zip › ijms-1563783-supplementary.pdf]

Supplementary Materials: Figure S1: Sectioning protocol for the collection of immunohistochemical marking and counting of cone photoreceptors; S2 Composite Image of cone-stained retina. Complete histochemistry protocol.

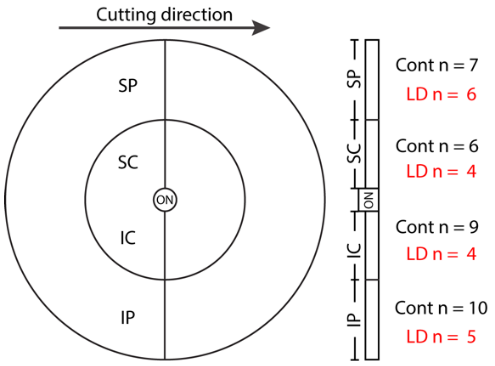

Figure S1. Sectioning protocol for immunocytochemistry. Serial sections of the vertically aligned eye were taken until the optic nerve was reached maintaining dorsal/ventral orientation. Some sections exhibited non-uniform staining across all of the four retinal quadrants of interest. For example, one retinal section may have exhibited irregular staining on the ventral half of the retina, but uniform, regular staining on the dorsal half. Only quadrants uniformly stained were included and those numbers are given to right.

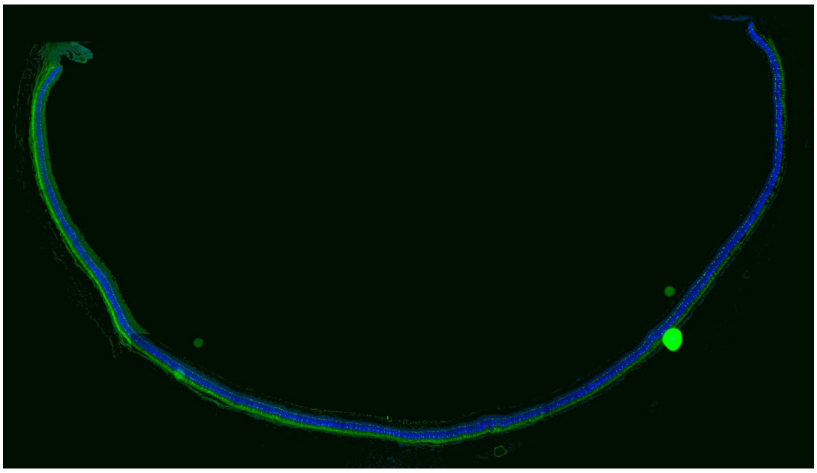

Figure S2. Composite of fluorescence photographs of 10μM-thick frozen retinal sections from a control rat. Sections were stained with a mCAR antibody and DAPI. Photographs were taken at 10X magnification. Total cone counts taken across such a section (at higher magnification) numbered 400-650 cones.

Detailed Protocol for Immunohistochemistry

Solutions: 2% Goat Serum/Phosphate-Buffered Saline (GS/PBS) – 9.8mL PBS + .2mL Goat Serum 20% GS/PBS Block Solution – 2.4mL PBS + 0.6mL Goat Serum (divided among 2 vials)

1° Antibody Mixture (1:500 Dilution) – 499μL 2% GS/PBS + 1μL 1° Antibody

2° Antibody Mixture (1:200 Dilution) – 995μL 2% GS/PBS + 5μL 2° Antibody

DAPI Mixture (1:1000 Dilution) – 1998μL PBS + 2μL DAPI (divided among 2 vials)

*Volumes of mixtures will change according to individual experiments. The volumes above are for 10 slides (5 controls, 5 experimental).

1. Turn slide warmer on. Go get a cooler of ice.
2. Label slides with a pencil (acetone in the next step will make ink run). Label the experiment, date, and what solutions will go on the slide (ex: 1°: R∝mCAR 1:500, 2°: G∝R-Alexa 1:200, DAPI).
3. Pour acetone into glass slide holder in the fume hood. Carry the container to the bench, and place slides into container for 3 minutes.
4. Remove slides, dry the back with a kimwipe, and place on the slide warmer at 50° C for one hour.
5. During the hour, make solutions and humidified chamber:
    a. Get 6 eppendorf tubes and label them B (for block), B (for block), 1°, 2°, D (for DAPI), and D (DAPI).
    b. Get one 15mL eppendorf tube and label it 2% GS/PBS.
    c. Get one tube of GS from freezer to let thaw.
    d. Make all of the solutions listed above, place them inside the cooler, and place the lid on the cooler (note: avoid any light exposure to 2° and to DAPI).
    e. Place remaining 2% GS/PBS into a 10mL syringe:
        i. Open syringe and .2μm filter. Remove plunger, and screw on filter.
        ii. Pour 2% GS/PBS into plunger, replace the plunger.
        iii. Turn upside down, unscrew filter, and push out excess air.
        iv. Screw filter back on, and place in cooler.
    f. Keep cooler lid ON so that light does not bleach the mixtures!
    g. Make humidified chamber:
        i. Place a folded paper towel in the bottom of a rectangular Tupperware container.
        ii. Wet the towel with DI water, drain any excess standing water out of the container, and place a square of parafilm on top of the paper towel (do not stretch the parafilm).
        iii. Place lid on Tupperware and wait for end of 1-hour timer.
6. Use a Pap Pen liquid blocker to draw an oval around the sections on each slide. Make oval close to sections, leaving a little extra room on top and bottom for application of liquid and vacuuming. Place in humidified chamber. Turn slide warmer off.
7. Centrifuge 20% block solution at 10k rpm for two minutes, and place .3mL of solution onto each slide. Cover and let sit in the humidified chamber for 1 hr (blocking solution blocks hydrophobic spots on tissue so that you don't get any false negatives).

    *For autofluorescence control, simply wash with PBS 3x (NO block). Put a drop of Polymount and place coverslip on slide. Place FLAT in a storage box in refrigerator until ready to be viewed.
8. Centrifuge 1° antibody mixture at 10k rpm for two minutes. Vacuum off the block solution, and place 95μL of the 1° antibody mixture onto experimental slides only, being careful to avoid the pellet at the bottom of the tube. Place 3-4 drops of 2% GS/PBS on the control slide(s). Close lid to humidified chamber, and let sit for 1 hour.
9. Vacuum off 1° antibody mixture (or 2% GS/PBS off the control) and immediately place 3-5 drops of filtered 2% GS/PBS onto the slide. Let this wash sit for five minutes before vacuuming off. Wash each slide 2 more times for a total of 3 washes.

    *after 3rd rinse, remove 2° to thaw a little. Vortex and centrifuge at 10k rpm for 2 minutes.
10. After the third wash, vacuum the 2% GS/PBS solution off, and place 95μL of the 2° antibody dilution onto each slide, including the control(s), and replace the cover to the humidified chamber. Cover the humidified chamber with a box to block light, and let sit for one hour.
11. Vacuum off 2° antibody and wash each slide three times with 2% GS/PBS as before.

12. After 3rd wash, take the 2% GS/PBS syringe to the sink, and pour solution out. Pour PBS into syringe, replace the filter and run a little through the syringe to get flush any remaining protein from the 2% GS/PBS solution.
13. Centrifuge the DAPI mixture at 10k rpm for two minutes. Vacuum off the 3rd wash, and add 200μL of the DAPI mixture to each slide, being careful to avoid the pellet at the bottom of the tube. Let the slides sit for five minutes.
14. Vacuum and wash each slide three times with filtered PBS (note: washes are immediate, not 5 minutes).
15. Vacuum off the final wash of PBS and place a drop of Poly/Mount onto the slide. Place cover slip on slide, and place slides into a flat slide tray. Put slide tray in fridge until ready to be viewed.
